# Incidence of HIV and Syphilis among Men Who Have Sex with Men (MSM) in Beijing: An Open Cohort Study

**DOI:** 10.1371/journal.pone.0138232

**Published:** 2015-10-01

**Authors:** Guowu Liu, Hongyan Lu, Juan Wang, Dongyan Xia, Yanming Sun, Guodong Mi, Liming Wang

**Affiliations:** 1 Beijing Municipality Center for Disease Control and Prevention, Beijing, China; 2 Beijing Ditan Hospital, Capital Medical University, Beijing, China; 3 National Center for AIDS/STD Control and Prevention, Chinese Center for Disease Control and Prevention, Beijing, China; Emory University's RSPH, UNITED STATES

## Abstract

**Background:**

This study investigated HIV and syphilis incidence among men who have sex with men (MSM) in Beijing, China.

**Methods:**

An open cohort was established from September 2009 to April 2012. Participants were followed up with every three to four months after recruitment and for thirty-one months in total. Chi-square tests were used to compare demographic and behavioral characteristics between participants who were followed up with and those lost to follow up. Univariate and multivariate Cox proportional hazards regression analyses were used to examine demographic and behavioral associations with HIV and syphilis incidence.

**Results:**

69.7% (699/1,003) of the participants finished at least two follow-up surveys during the study period. Variables which corresponded to increased loss to follow-up included younger age, less education, non-identification of homosexual identity, and migrant status. A total of 1,045 person-years (PYs) and 1,016.4 PYs were followed up for HIV and syphilis incidence estimation, respectively. The HIV incidence was 5.9 per 100 PYs and 7.8 per 100 PYs for syphilis. The predictors for the high HIV incidence included unsafe anal sex, sex after drinking alcohol and STI infection.

**Conclusion:**

HIV incidence increased rapidly within the cohort, but syphilis incidence remained stable and decreased. More research is needed to provide multi-pronged HIV prevention interventions among MSM in order to reduce the increasing burden of HIV and sexually transmitted infections (STIs) in China.

## Introduction

Unsafe sex among men who have sex with men (MSM) has increasingly become a major route of Human Immunodeficiency Virus (HIV) transmission in China. By 2011, 17.4% of the estimated cumulative 780,000 cases of people living with HIV/AIDS were infected through same-gender sex and MSM accounted for 29.4% of the estimated 48,000 newly reported HIV cases in that year. A recent survey among MSM in four Chinese cities indicates that the HIV/AIDS prevalence among this population is approximately 5.0% of which a large proportion are newly infected cases [1].

As a lifestyle and orientation, homosexuality is largely unacceptable in Chinese society, despite growing tolerance in certain realms of academia and study. It is unsurprising that the hard to reach population size of MSM in China is difficult to ascertain due to multiple factors, including nationwide stigma and discrimination. A report published collaboratively by Chinese sociologists and psychologists in 2005 estimated the number of MSM in China to be at least 30 million, while some public health professionals conjecture that the MSM population size is between 5 million and 10 million [2].

As a sprawling mega-city, Beijing, China sees a massive influx of MSM every year [3]. Several data sources, including sentinel surveillance and specific surveys indicate that homosexual transmission has become the dominant route of HIV transmission in China’s capital[4]. MSM as a proportion of China’s HIV-diagnosed population have increased from 3.7% in 2005 to 10.2% in 2010 [5]. In order to further understand the HIV epidemic among MSM in Beijing, we initiated an open cohort in 2009 to investigate the incidence of HIV and syphilis prospectively. Resulting evidence will be used to identify target population for prioritized HIV prevention, to recommend more strategic resource allocation, and to further assess need within the demographic.

## Methods

### Ethics Statement

All participants granted with written informed consent to participate in this study. Informed consent was explained to all participants by trained study staff and only those who agreed to sign the terms for participation were enrolled. All of the signed study documents were kept in a secured cabinet accessible only by authorized study staff at Beijing Municipality Center for Disease Control and Prevention (Beijing CDC). The study protocol and informed consent procedures were reviewed and approved by the ethics committee of Beijing CDC.

### Overall Study Design

We conducted an open cohort enrollment of MSM in Beijing from September 1^st^, 2009 until April 30^th^, 2012. Participants were followed up every three to four months after recruitment. At the enrollment, participants’ demographic, behavioral and HIV as well as syphilis status information were collected by trained interviewers through a questionnaire based survey. A similar follow-up questionnaire was completed at each subsequent visit.

The demographic questionnaire assessed age, education level, marital status, monthly income, and self-reported sexual orientation etc. Behavioral data included types of sexual activities conducted, frequency of condom use during sexual activity, numbers of sexual partners, recreational drug use, and alcohol consumption as well as HIV and syphilis testing history. At both enrollment and each follow up visit, blood was drawn for serologic testing for HIV and syphilis.

### Study population and Setting

The target population of our study was MSM currently living in Beijing. MSM were defined as males who reported having ever had sex with another man. Eligible criteria for study enrollment included: 1) HIV and syphilis negative status; 2) 17 years of age and older; 3) having anal or oral sex activity with another male in the past 4 months, and 4) willingness to participate in the study with written informed consent.

### Sampling and Recruitment

Three rounds of enrollment were introduced during the open cohort study period in order to keep participant numbers relatively consistent and stable over time. The first round of recruitment was from September 1^st^ to October 30^th^ 2009, using the respondent-driven sampling (RDS) which has been described in previous cohort studies in Beijing [6].

**Fig 1:**
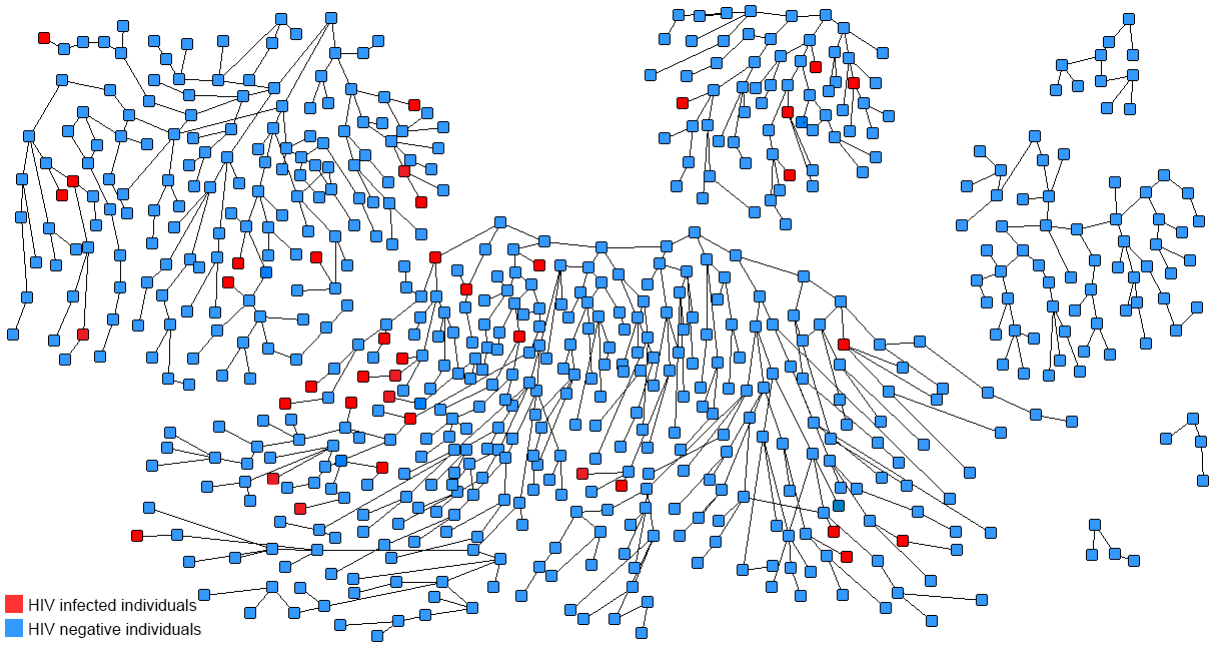
Recruitment Tree of the first round of enrollment using the respondent-driven sampling (RDS) method.

In this study, seven initial “seeds” were chosen to initiate RDS. These “seeds” were recruited through multiple strategies including advertisement in the gay-website www.bf99.com, outreach by a nongovernmental AIDS volunteer group and referrals from sexual transmitted infections (STIs) clinics. Among these 7 seeds, the median age was 31 years old; 4 were not official Beijing residents and 5 had a college degree or above. Each seed was trained to recruit 3 possible eligible members to the study from the target population using a referral card with a unique number. A successfully enrolled individual could be anonymously linked to the index seed. After 17 waves of recruitment, the respondent driven sample was considered stabilized. Respondents were given 50 RMB (less than $8 USD) for each completed survey as compensation for transportation and meals. Fig 1 shows the recruitment tree of the first round of enrollment in our study using the RDS method.

The second and third rounds of recruitment were conducted in the same months (September through October) of the years 2010 and 2011. All the participants were recruited from a volunteer HIV testing and counseling (VCT) site of Beijing. To reduce the impact of becoming lost-follow-up, the distribution of demographic characteristics of participants recruited in the second and third rounds was matched with those who were enrolled in the first round but lost follow up. Lost-follow-up was defined as showing up only once or not at all at scheduled follow up visits.

Behavioral survey and laboratory tests of HIV and syphilis were performed every 3 months by trained interviewers during the study period. HIV-positive cases were referred to the National HIV/AIDS Care Program for further care and free anti-retroviral (ARV) treatment, while positive syphilis cases were referred to designated local hospitals for discounted treatment.

### Statistical Methods

SAS Version 9.2 (Cary, North Carolina, USA) for Window X64 was used for data analysis. Categorical variables were reported in both numbers and percentages, while continuous variables were presented as “mean ± standard deviation”. Chi-square tests were used to compare demographic and behavioral characteristics between participants who were followed up with and those lost to follow up. Since it was impossible to know the exact date of HIV sero-conversion among cohort participants, the date midway between the last negative and the first positive tests was assumed to be the HIV infection date in our analysis. Univariate and multivariate Cox proportional hazards regression analyses were used to examine demographic and behavioral associations with HIV and syphilis incidence. Factors with *P* ≤0.05 in the univariate analysis were included in a stepwise Cox multiple regression model with entry criteria of *P*<0.2 and exit of *P*>0.05. A *P* value of 0.05 or less was considered statistically significant.

### Laboratory Tests

HIV infection status was screened by an HIV-1/2 Antigen/Antibody combo enzyme immunoassay (Beijing Wantai Biological Medicine Company, China). Repeatedly reactive samples were determined by an HIV-1/2 Western blot (HIV Blot 2.0 MPDiagnostics, Singapore). Syphilis was screened by an enzyme immunoassay (Zhuhai Lizhu Company, China) and a rapid plasma regain test (Shanghaikehua Companly, China). Confirmation of syphilis infection diagnosis was established using the Treponema pallidum particle assay (FujirebioDiagnostics, Inc, Japan).

## Results

**Fig 2:**
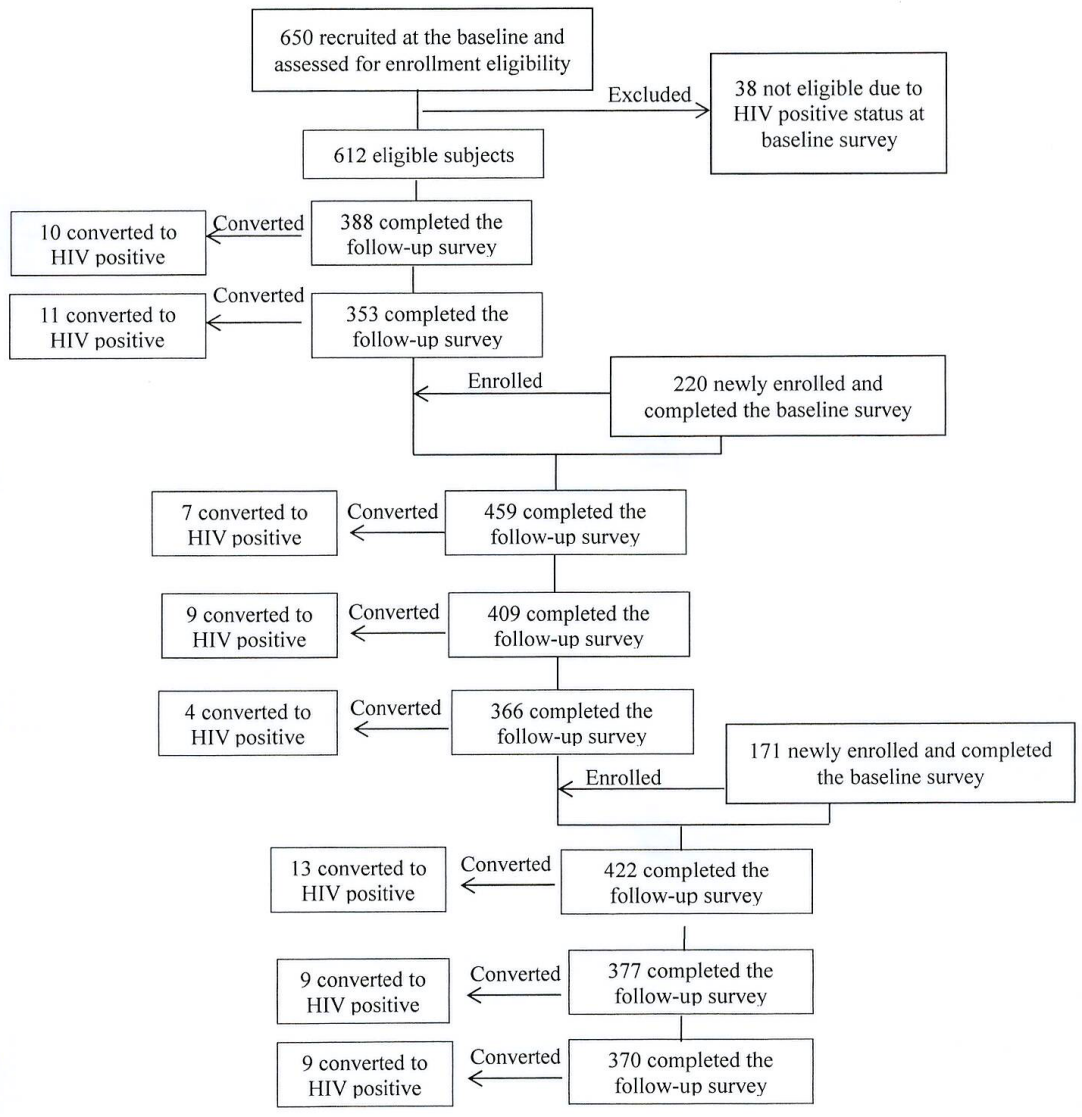
Participation of eligible men who have sex with mem in the open cohort study in Beijing, 2009–2012.

During the study period, 1,041 MSM were surveyed, among whom 1,003 baseline HIV- negative MSM were recruited in the open cohort. 612 participants were recruited through RDS, while 220 and 171 from the second and third round were recruited from the VCT site mentioned above. In total, eight sessions of follow up visits were conducted. Among 1,003 participants, 218 completed all eight follow up visits. 699 finished at least two surveys during the study period. These 699 participants were included in the final data analysis for incidence estimation. 304 participants were lost-to-follow-up. Fig 2 shows the participation of subjects in our study at baseline and at each follow-up visit.

### Characteristics of the study participants

At enrollment, as shown in Table 1, the majority of participants were relatively young with a median age of 28.0 (IQR: 24.0–35.0) years old. More than 40% of participants had completed college and/or post-graduate studies. Approximately three quarters of participants had been residing in Beijing for more than 24 months and 58.0% reported their monthly income was greater than 2,000 RMB. Nearly 70% of participants self-identified as homosexual at enrollment while 19.2% were married to women. Participants lost to follow up were much younger (*P*<0.05), less educated (*P*<0.05), more likely to be migrants (*P*<0.05) and did not self-identify as homosexual (*P*<0.05).

**Table 1:** Baseline characteristics of Men who have sex with men (MSM) participants by followed-up status, Beijing, 2009–2012.

Characteristic	Total N = 1003	Follow-up N = 699(%)	Lost-to-follow-up N = 304(%)	Value	*P* value
Age (years)					
17–24	288(28.7)	169(24.2)	119(39.1)	χ2 = 24.2	<0.05
25–29	273(27.3)	204(29.2)	69(22.7)		
30–34	181(18.0)	131(18.7)	50(16.5)		
35–39	115(11.4)	89(12.7)	26(8.6)		
40–88	146(14.6)	106(15.2)	40(13.2)		
Education					
Less than senior middle school	287(28.6)	177(25.3)	110(36.2)	χ2 = 17.3	<0.05
Senior Middle school	305(30.4)	208(29.8)	97(31.9)		
College degree and above	411(41.0)	314(44.9)	97(31.9)		
How long have you resided in Beijing (months)					
3–11	139(13.9)	66(9.4)	73(24.0)	χ2 = 45.8	<0.05
12–24	106(10.6)	65(9.3)	41(13.5)		
25-	758(75.5)	568(81.3)	190(62.5)		
Monthly income (Yuan)					
<1000	154(15.4)	104(14.9)	50 (16.4)	χ2 = 6.4	0.09
1000-	267(26.6)	173(24.8)	94(30.9)		
2000-	254(25.3)	179(25.6)	75(24.7)		
3000-	328(32.7)	243(34.8)	85(28.0)		
Marital status					
Single	715(71.3)	499(71.4)	216(71.1)	χ2 = 0.0	0.99
Married	193(19.2)	134(19.2)	59(19.4)		
Divorced/widowed	95(9.5)	66(9.4)	29(9.5)		
Sexual orientation					
Homosexuality	693(69.1)	506(72.4)	187(61.5)	χ2 = 15.4	<0.05
Heterosexuality	9(0.9)	3(0.4)	6(2.0)		
Bi-sexuality	301(30.0)	190(27.2)	111(36.5)		
HIV testing history					
Ever tested	633(63.1)	498(71.2)	135(44.4)	χ2 = 65.5	<0.05
Never tested	370(36.9)	201(28.8)	169(55.6)		
Sexually transmitted infections (STI) testing history					
Ever had STI diagnosed	159(15.8)	124(17.7)	35(11.5)	χ2 = 6.2	0.01
Never diagnosed to have STI	844(84.2)	575(82.3)	269(88.5)		
Ever had circumcision surgery					
Yes	80(8.0)	63(9.0)	17(5.6)	χ2 = 3.4	0.07
No	923(92.0)	636(91.0)	287(94.4)		
Age at first same-gender sex	20.0(18–22)	20.0(18.0–22.0)	19.0(17.0–22.0)	Z = -2.2	0.03
Engaging in group sex in past 6 months					
Yes	119(11.9)	86(12.3)	33(10.9)	χ2 = 0.4	0.52
No	884(88.1)	613(87.7)	271(89.1)		
Engaging in unsafe vaginal sex in past 6 months					
Yes	144(14.4)	84(12.0)	60(19.7)	χ2 = 10.3	<0.05
No	859(85.6)	615(88.0)	244(80.3)		
Engaging in unsafe anal sex in past 6 months					
Yes	573(57.1)	416(59.5)	157(51.6)	χ2 = 5.4	0.02
No	430(42.9)	283(40.5)	147(48.4)		

Approximately 63% of participants at baseline had ever tested for HIV and 15.8% had been previously diagnosed with STIs. Only 8.0% stated a history of circumcision surgery. The median age of first homosexual contact was 20.0 (IQR: 18.0–22.0) years old. More than half (57.1%) reported engaging in unprotected anal sex and 14.4% had vaginal sex in the past six months. Compared with respondents retained in the cohort, those lost to follow-up were more likely to have had vaginal sex (19.7% vs. 12.0%, *P*<0.05), less likely to have had unprotected anal sex (51.6% vs. 59.5%, *P* = 0.02), less likely to have an HIV testing history (44.4% vs. 71.2%, *P*<0.05) and less likely to have ever been diagnosed with an STI (11.5% vs.17.7%, *P* = 0.01).

### HIV and syphilis incidence and associated risk factors

During the study period, 62 HIV seroconversions were reported over a total of 1,045.7 person-years (PYs) of observation, resulting in an incidence rate of 5.9 per 100 PYs (95% confidence interval (CI): 4.6–7.6). The HIV incidence was 5.9 (95% CI: 3.9–8.8), 5.7 (95% CI: 3.7–8.7), and 6.0 (95% CI: 4.2–8.4) per 100 person-years in the first, second and third year of the study.

A total of 690 respondents for syphilis incidence estimation contributed 1,016.4 PYs of follow up data. There were 79 newly diagnosed syphilis cases, yielding the syphilis incidence rates of 7.8 per 100 PYs (95% CI: 6.2–9.7). Syphilis incidence was 10.5 (95% CI: 7.2–15.2), 11.2 (95% CI: 7.9–15.7) and 6.9 (95% CI: 4.7–10.1) per 100 PY in the first, second and third year of the study, respectively.

In the multivariable analysis of risk factor assessment for HIV sero-conversion (Table 2), participants aged 17–24 years old saw the highest HIV incidence at 8.5 per 100 PYs (95% CI: 5.4–13.4), although the difference was not statistically significant. Participants with an education background of junior high school or less had the highest HIV incidence of 8.8 per 100 PYs (95% CI: 5.8–13.2). This was significantly higher (*P* = 0.01) than participants with senior high school or an equivalent educational background (HIV incidence of 4.3, 95% CI: 2.6–7.3). Those who engaged in unprotected anal sex during the past 4 months were 2.1 (95% CI: 1.3–6.9) times more likely to become infected with HIV after adjustment for other factors. Participants who had sex after drinking alcohol in the past 4 months were more likely to become infected with HIV (adjusted hazard ratio (aHR) of 1.9 (95% CI: 1.1–4.2)). Participants who had been diagnosed with STI infection in the past 4 months were 4.6 (95% CI: 1.6–13.6) times more likely to become infected with HIV than those without a previous STI.

**Table 2:** HIV incidence by characteristics in followed-up men who have sex with men (MSM), Beijing, 2009–2012.

Characteristic	Number of followed up N (%)	Number of Sero-converted n (%)	Observed person-years (PY)	Incidence Per 100 PY (CI)^§^	Unadjusted HR^+^ (CI) ^§^	Adjusted HR^+^ (CI) ^§^	*P* value
Total	699	62	1045.7	5.9 (4.6–7.6)	---	----	*---*
Age (years) at baseline							
17–24	169(24.2)	19(30.6)	222.4	8.5(5.4–13.4)	1.0	1.0	
25–29	204(29.2)	17(27.4)	298.7	5.7(3.5–9.2)	0.7(0.4–1.3)	0.6(0.3–1.2)	0.17
30–34	131(18.7)	13(21.0)	210.5	6.2(3.6–10.6)	0.8(0.4–1.5)	0.5(0.2–1.1)	0.11
35–39	89(12.7)	6(9.7)	140.8	4.3(1.9–9.5)	0.5(0.2–1.3)	0.4(0.1–1.1)	0.07
40–88	106(15.2)	7(11.3)	173.3	4.0(1.–8.5)	0.5(0.2–1.1)	0.4(0.1–1.0)	0.06
Education at baseline							
Junior high school and less	177(25.3)	23(37.1)	261.6	8.8(5.8–13.2)	1.0	1.0	
Senior high school or equivalent	208(29.8)	14(22.6)	322.9	4.3(2.6–7.3)	0.5(0.3–0.9)	0.4(0.2–0.8)	0.01
College degree and above	314(44.9)	25(40.3)	461.2	5.4(3.6–8.0)	0.6(0.4–1.1)	0.6(0.3–1.1)	0.12
How long have you resided in Beijing (months) at baseline							
3–11	66(9.4)	7(11.3)	87.3	8.0(3.8–16.8)	1.0	1.0	
12–24	65(9.3)	7(11.3)	98.6	7.1(3.4–14.9)	0.9(0.3–2.6)	1.0(0.3–3.1)	0.95
25-	568(81.3)	48(77.4)	859.8	5.6(4.2–7.4)	0.7(0.3–1.6)	1.0(0.4–2.4)	1.00
Monthly income (Yuan) at baseline							
<1000	104(14.9)	8(12.9)	157.1	5.1(2.5–10.2)	1.0	1.0	
1000-	173(24.8)	20(32.3)	278.0	7.2(4.6–11.2)	1.4(0.6–3.2)	1.8(0.7–4.3)	0.19
2000-	179(25.6)	17(27.4)	261.9	6.5(4.0–10.4)	1.3(0.5–2.9)	1.9(0.8–4.8)	0.16
3000-	243(34.8)	17(27.4)	348.8	4.9(3.0–7.8)	0.9(0.4–2.2)	1.4(0.6–3.7)	0.45
Marital status at baseline							
Single	499(71.4)	46(74.2)	721.0	6.4(4.7–8.5)	1.0	1.0	
Married	134(19.2)	14(22.6)	203.6	6.9(4.1–11.6)	1.1(0.6–2.0)	1.5(0.7–3.4)	0.33
Divorced/widowed	66(9.4)	2(3.2)	121.2	1.7(0.4–6.6)	0.3(0.1–1.1)	0.4(0.1–2.0)	0.29
Sexual orientation at baseline							
Homosexuality	506(72.4)	46(74.2)	740.8	6.2(4.6–8.3)	1.0	1.0	
Heterosexuality	3(0.4)	0	3.4	0	---	---	---
Bi-sexuality	190(27.2)	16(25.8)	301.5	5.3(3.3–8.7)	0.9(0.5–1.5)	0.8(0.4–1.5)	0.47
HIV testing history at baseline							
Ever tested	498(71.2)	45(72.6)	771.1	5.8(4.4–7.8)	1.0	1.0	
Never tested	201(28.8)	17(27.4)	274.7	6.2(3.8–9.9)	1.0(0.6–1.8)	1.2(0.6–2.1)	0.63
Ever had circumcision surgery at baseline							
Yes	63(9.0)	6(9.7)	94.5	6.3(1.6–10.9)	1.0	1.0	
No	636(91.0)	56(90.3)	951.2	5.9(4.6–7.8)	0.9(0.5–2.8)	0.9(0.4–2.8)	0.86
Ever engaging in group sex at baseline							
No	613(87.7)	51(82.2)	919.8	5.5(4.2–7.3)	1.0	1.0	
Yes	86(12.3)	11(17.8)	125.9	8.7(4.8–15.8)	1.6(0.9–3.0)	1.5(0.7–2.9)	0.27
Engaging in unsafe vaginal sex since the last study visit*							
Yes	70(10.0)	6(9.7)	112.4	5.3(2.4–11.9)	1.0	1.0	
No	629(90.0)	56(90.3)	933.4	6.0(4.6–7.8)	1.1(0.5–2.6)	1.4(0.6–3.7)	0.45
Engaging in unsafe anal sex since the last study visit*							
No	40(5.7)	1(1.6)	56.3	1.8(0.9–13.7)	1.0	1.0	
Yes	659(94.3)	61(98.4)	989.4	6.2(4.7–7.8)	3.4(1.1–8.2)	2.1(1.3–6.9)	0.04
Drug use since the last study visit*							
No	693(99.1)	61(98.4)	1040.8	5.9(4.6–7.5)	1.0	1.0	
Yes	6(0.9)	1(1.6)	4.9	20.4(2.9–143.6)	3.3(0.5–24.1)	2.0(0.2–17.5)	0.53
Sex after drinking alcohol since the last study visit*							
No	637(91.1)	53(85.5)	970.3	5.5(4.2–7.2)	1.0	1.0	
Yes	62(8.9)	9(14.5)	75.5	11.9(6.2–22.9)	2.2(1.1–4.4)	1.9(1.1–4.2)	0.04
Diagnosed with sexually transmitted infections since the last study visit*							
No	687(98.3)	58(93.5)	1035	5.6(4.3–7.2)	1.0	1.0	
Yes	12(1.7)	4(6.5)	10.8	37.0(14.0–99.6)	6.2(2.2–17.3)	4.6(1.6–13.6)	0.01

* If the participant has answered “yes” at any of the follow up visits, then the variable is considered as “yes” during the analysis.

^**+**^ HR = Hazard Risk

^**§**^ CI = Confidence Interval.

In a similar multivariate analysis for syphilis incidence (Table 3), participants 40–88 years old (aHR 2.2, 95% CI: 1.0–5.0) had a higher risk of contracting syphilis with an incidence of 12.7 per 100 PYs compared with those 17–24 years with 7.8 cases per 100 PYs. In addition, recreational drug use during the past 4 months was shown to be statistically related to syphilis infection (aHR 11.7, 95% CI: 2.4–58.9). However, in our cohort, single MSM appeared to be negatively associated with increased syphilis incidence compared with married MSM (aHR 0.4, 95% CI: 0.2–0.9).

**Table 3:** Syphilis incidence by characteristics in followed-up men who have sex with men (MSM), Beijing, 2009–2012.

Characteristic	Number of followed up N (%)	Number of Sero-converted n (%)	Observed person-years (PY)	Incidence Per 100 PY (CI) ^§^	Unadjusted HR^+^ (CI) ^§^	Adjusted HR^+^ (CI) ^§^	*P* value
Total	690	79	1016.4	7.8 (6.2–9.7)	—	—	*—*
Age (years) at baseline							
17–24	166(24.1)	17(21.5)	218.8	7.8(4.8–12.5)	1.0	1.0	
25–29	204(29.6)	16(20.3)	293.3	5.4(3.3–8.9)	0.7(0.3–1.3)	0.7(0.3–1.4)	0.26
30–34	128(18.6)	13(16.5)	204.5	6.4(3.7–10.9)	0.7(0.4–1.5)	0.8(0.4–1.7)	0.55
35–39	87(12.6)	12(15.2)	133.9	9.0(5.1–15.8)	1.1(0.5–2.3)	1.3(0.6–3.1)	0.48
40–88	105(15.2)	21(26.5)	165.8	12.7(8.3–19.4)	1.5(0.8–2.8)	2.2(1.0–5.0)	0.05
Education at baseline							
Junior high school and less	175(25.4)	23(29.1)	256.5	9.0(5.9–13.5)	1.0	1.0	
Senior high school or equivalent	203(29.4)	24(30.4)	310.3	7.7(5.2–11.5)	0.9(0.5–1.6)	0.8(0.4–1.5)	0.45
College degree and above	312(45.2)	32(40.5)	449.6	7.1(5.0–10.1)	0.9(0.5–1.5)	0.9(0.5–1.8)	0.87
How long have you resided in Beijing (months) at baseline							
3–11	66(9.6)	8(10.1)	86.4	9.3(4.6–18.5)	1.0	1.0	
12–24	64(9.3)	5(6.3)	97.9	5.1(2.1–12.3)	0.5(0.2–1.5)	0.5(0.2–1.7)	0.28
25-	560(81.2)	66(83.5)	832.1	7.9(6.2–10.1)	0.8(0.4–1.7)	0.7(0.3–1.6)	0.45
Monthly income (Yuan) at baseline							
<1000	104(15.1)	11(13.9)	155.2	7.1(3.9–12.8)	1.0	1.0	
1000-	171(24.8)	25(31.7)	271.2	9.2(6.2–13.6)	1.3(0.6–2.6)	1.5(0.7–3.2)	0.31
2000-	176(25.5)	18(22.8)	254.1	7.1(4.5–11.2)	1.0(0.5–2.1)	1.3(0.6–2.9)	0.57
3000-	239(34.6)	25(31.6)	335.9	7.4(5.0–11.0)	1.1(0.6–2.4)	1.4(0.6–3.2)	0.40
Marital status at baseline							
Single	494(71.6)	56(70.9)	700.2	8.0(6.1–10.4)	1.0	1.0	
Married	131(19.0)	10(12.7)	199.9	5.0(2.7–9.3)	0.6(0.3–1.2)	0.4(0.2–0.9)	0.05
Divorced/widowed	65(9.4)	13(16.5)	116.3	11.2(6.5–19.2)	1.2(0.7–2.3)	0.8(0.4–1.6)	0.54
Sexual orientation at baseline							
Homosexuality	498(72.2)	61(77.2)	716.7	8.5(6.6–10.9)	1.0	1.0	
Heterosexuality	3(0.4)	0	3.4	---	---	---	---
Bi-sexuality	189(27.4)	18(22.8)	296.3	6.0(3.8–9.6)	0.7(0.4–1.1)	0.9(0.4–1.5)	0.59
HIV testing history at baseline							
Ever tested	489(70.9)	57(71.1)	743.7	7.7(5.9–9.9)	1.0	1.0	
Never tested	201(29.1)	22(27.9)	272.7	8.1(5.3–12.3)	1.1(0.7–1.8)	1.1(0.7–1.8)	0.74
Ever had circumcision surgery at baseline							
Yes	62(9.0)	5(6.3)	93.1	5.4(2.2–12.9)	1.0	1.0	
No	628(91.0)	74(93.7)	923.3	8.0(6.4–10.1)	1.4(0.6–3.5)	1.3(0.5–3.4)	0.55
Ever engaging in group sex at baseline							
No	609(88.3)	70(88.6)	897.7	7.6(3.9–14.6)	1.0	1.0	
Yes	81(11.7)	9(11.4)	118.7	7.8(6.2–9.9)	1.0(0.5–2.1)	1.0(0.5–2.1)	0.94
Engaging in unsafe vaginal sex since the last study visit*							
Yes	70(10.1)	4(5.1)	111.2	3.6(1.3–9.6)	1.0	1.0	
No	620(89.9)	75(94.9)	905.2	8.3(6.6–10.4)	2.5(1.0–6.9)	1.8(0.6–5.4)	0.29
Engaging in unsafe anal sex since the last study visit*							
No	40(5.8)	3(3.8)	57.0	5.3(1.7–16.3)	1.0	1.0	
Yes	650(94.2)	76(96.2)	959.4	7.9(6.3–9.9)	1.5(0.5–4.6)	1.6(0.5–5.0)	0.46
Drug use since the last study visit*							
No	685(99.3)	77(97.5)	1012.7	7.6(6.1–9.5)	1.0	1.0	
Yes	5(0.7)	2(2.5)	3.7	53.7(13.4–214.6)	11.3(2.7–47.4)	11.7(2.4–58.9)	<0.05
Diagnosed with sexually transmitted infections since the last study visit*							
No	631(91.5)	72(91.1)	944.0	7.6(6.1–9.6)	1.0	1.0	
Yes	59(8.6)	7(8.9)	72.4	9.6(4.6–20.3)	1.3(0.6–3.0)	1.2(0.5–2.8)	0.66

* If the participant has answered “yes” at any of the follow up visits, then the variable is considered as “yes” during the analysis.

^**+**^ HR = Hazard Risk

^**§**^ CI = Confidence Interval.

## Discussion

Our study demonstrated that HIV incidence among MSM from September 2009 through April 2012 in Beijing was 5.9 per 100 PYs, a drastic increase from 2.9% in 2005, 3.6% in 2006 [7] and 2.6% in 2007 [8]. Recent mathematical modeling projected that the HIV epidemic will continue to increase among MSM until 2020 in Beijing [9]. HIV incidence in this study was lower than other reports [10,11]. One cohort study following 797 seronegative MSM in Beijing enrolled from August to December 2009 showed an HIV incidence of 8.09 per 100 PYs at 12-months’ follow up [10]. Another prospective cohort study conducted among Beijing MSM from September to October 2009 and followed for eight months, had an HIV incidence of 7.83 per 100 PYs [11]. Our study had, in comparison, an extensive follow-up period of up to 31 months for open cohort, thus conferring increased reliability.

The syphilis incidence of 7.8 per 100 PYs (95% CI: 6.2–9.7) in our study was similar to that of two other cohort studies just mentioned above, which were 5.9 per 100 PYs (95% CI: 5.44–6.40) [10] and 11.11 per 100 PYs (95% CI: 6.8–17.8) [11], respectively. Syphilis incidence seemed to decrease compared with data from a Beijing cohort study from 2006–2007 which found an incidence rate of 16.9% per 100 PYs (95% CI: 12.4–21.3) [8,12]. Explanations for the decline are not entirely clear, and further studies are needed to address this issue.

In this open cohort study, engaging in unprotected anal sex, having sex after drinking alcohol and getting infected with STIs were risk factors for HIV infection. High risk sexual behaviors, especially unsafe anal sex, were considered to be the main reason for HIV transmission among MSM. 94% (659/699) of the participants reported having unsafe anal sex during the 31-month follow-up period. This was much higher than a recent meta-analysis indicating that 63.7% of MSM in China had ever had unprotected anal intercourse during the 6 months prior to recruitment [13]. Drinking alcohol is likely associated with HIV infection in MSM due to lowered inhibitions while under the influence of alcohol [14,15].

Among the 699 participants included in analysis, only 8.9% (62/699) reported drinking alcohol prior to engaging in sexual intercourse in the last 4 months. This was much lower compared to other studies conducted among MSM in Beijing (e.g. reported sex after alcohol use within a three-month period of 42.1% [16], or reported sex after alcohol use within a one-year time span of 57.9% [17]). This inconsistency may be due to different instruments and research designs. Nonetheless, our study and former studies identified a significant association between HIV infection and alcohol intake prior to sexual activity. The finding may point to more strategic intervention approaches needed to reduce HIV transmission among Chinese MSM, a significant target population which has typically seen little public health attention. In addition, having STIs is another risk factor for HIV infection due to biological and behavioral links between STIs and HIV, as confirmed by former studies [18–20]. However, according to a survey carried out among young MSM in colleges in Beijing, less than two-thirds (63.1%) knew that STIs could facilitate HIV infection [21]. Therefore, it is also recommended that future HIV prevention interventions should address this awareness gap.

During this open cohort study period, 69.7% (699/1,003) of the participants had at least two follow-ups. The retention rate in our study was lower than two previous cohort studies (one year retention rate was 86.2% in 2007 and 86.8% in 2009) [8,10] but higher than one other study (8-month retention rate was 54.6%) [11] among MSM in Beijing. Of note is that the study duration of these three previous studies was only one year or less, while we followed participants for up to 31 months. Participants lost to follow up were younger, less educated, more likely to migrate, and less likely to self-identify as homosexual, which is consistent with previous studies [11,22,23]. Higher loss to follow-up may also be due to varying work or schooling schedules affecting migration patterns and general accountability [24]. Those less educated tend to have temporary employment and are therefore too mobile to be retained in the cohort or in HIV care. Moreover, some cross-sectional studies in China demonstrated that compared to local MSM with permanent residency, migrant MSM engage in more high-risk sexual behaviors [25,26], and have higher HIV and syphilis incidence rates[10,11]. Thus, taking all of these characteristics into consideration, the incidence of HIV and syphilis may be underestimated in our study. Future studies should consider employing different methods for increasing retention of these population subgroups in HIV treatment and care.

Findings in this study may have several limitations. First, our data are hampered by the lack of reliable population-based measurements of MSM in Beijing. Secondly, the majority of MSM in Beijing are domestic and international migrants which may prevent having a more representative cohort that accurately reflects the city’s HIV and syphilis epidemics.

This study also has several strengths and implications. First, it was an open cohort and had a relatively long follow-up period compared to previous studies. Second, the high HIV incidence and prevalence found among MSM in Beijing indicated that the epidemic in this group is extremely serious and that effective targeted interventions are urgently required. Third, it is imperative that young, migrant and less educated MSM receive more attention from public health providers to improve their long-term engagement in HIV treatment and care. Finally, HIV education gaps regarding the association between STIs and alcohol use with HIV infection should be addressed via future health promotion programs.

## Conclusions

The HIV and syphilis incidence rates were 5.9 per 100 PYs and 7.8 per 100 PYs, respectively, in Beijing, China from 2009 to 2012. Significant predictors of high HIV incidence included unsafe anal sex, having sex after drinking alcohol and previous STIs infection. More research is needed to provide multi-pronged HIV prevention interventions among MSM in order to reduce the increasing burden of HIV and STIs in China.
